# Whites’ County-Level Racial Bias, COVID-19 Rates, and Racial Inequities in the United States

**DOI:** 10.3390/ijerph17228695

**Published:** 2020-11-23

**Authors:** Marilyn D. Thomas, Eli K. Michaels, Sean Darling-Hammond, Thu T. Nguyen, M. Maria Glymour, Eric Vittinghoff

**Affiliations:** 1 Department of Epidemiology and Biostatistics, School of Medicine, University of California, 550 16th St 2nd floor, San Francisco, CA 94158, USA; Maria.Glymour@ucsf.edu (M.M.G.); Eric.Vittinghoff@ucsf.edu (E.V.); 2 Department of Psychiatry, School of Medicine, University of California, 1001 Potrero Ave, San Francisco, CA 94110, USA; 3 Division of Epidemiology, School of Public Health, University of California, 2121 Berkeley Way, Room 5302, Berkeley, CA 94720, USA; elikmichaels@berkeley.edu; 4 Goldman School of Public Policy, University of California, 2607 Hearst Ave, Berkeley, CA 94720, USA; sean.darling.hammond@berkeley.edu; 5 Department of Family and Community Medicine, School of Medicine, University of California, 995 Potrero Ave, San Francisco, CA 94110, USA; Thu.Nguyen@ucsf.edu

**Keywords:** COVID-19, health inequities, racism and discrimination, social determinants of health

## Abstract

Mounting evidence reveals considerable racial inequities in coronavirus disease 2019 (COVID-19) outcomes in the United States (US). Area-level racial bias has been associated with multiple adverse health outcomes, but its association with COVID-19 is yet unexplored. Combining county-level data from Project Implicit on implicit and explicit anti-Black bias among non-Hispanic Whites, Johns Hopkins Coronavirus Resource Center, and *The New York Times*, we used adjusted linear regressions to estimate overall COVID-19 incidence and mortality rates through 01 July 2020, Black and White incidence rates through 28 May 2020, and Black–White incidence rate gaps on average area-level implicit and explicit racial bias. Across 2994 counties, the average COVID-19 mortality rate (standard deviation) was 1.7/10,000 people (3.3) and average cumulative COVID-19 incidence rate was 52.1/10,000 (77.2). Higher racial bias was associated with higher overall mortality rates (per 1 standard deviation higher implicit bias b = 0.65/10,000 (95% confidence interval: 0.39, 0.91); explicit bias b = 0.49/10,000 (0.27, 0.70)) and higher overall incidence (implicit bias b = 8.42/10,000 (4.64, 12.20); explicit bias b = 8.83/10,000 (5.32, 12.35)). In 957 counties with race-specific data, higher racial bias predicted higher White and Black incidence rates, and larger Black–White incidence rate gaps. Anti-Black bias among Whites predicts worse COVID-19 outcomes and greater inequities. Area-level interventions may ameliorate health inequities.

## 1. Introduction

Mounting evidence reveals considerable racial inequities in coronavirus disease 2019 (COVID-19) incidence and mortality rates in the United States (US) [1,2,3,4,5,6]. Structural racism—defined as ongoing interactions between macro-level systems, social forces, institutions, and ideologies that constrain the opportunities, resources, and power of minoritized racial groups—has been implicated as a fundamental cause of COVID-19 inequities [7,8]. Structural racism governs the distribution of a broad range of health-promoting resources that make it much more difficult for minoritized populations to access preventive care and avoid high-risk exposures [9,10,11,12,13]. For instance, residential segregation may increase the likelihood that racial/ethnic minoritized groups live in densely populated areas with substandard and crowded housing conditions [1,14,15] that make social distancing practices more difficult. Thus, historical and ongoing structural racism likely drives the community spread of COVID-19 and the unequal burden of poor outcomes across racial/ethnic groups [1,5,16,17,18].

Area-level racial bias has been described as a “cognitive manifestation of structural racism” [19], which undergirds institutional and interpersonal forms of discrimination [20]. At the individual level, racial bias is defined as preconceived beliefs, attitudes, and expectations about members of a racial group [10], which can influence racial discrimination in day-to-day interactions and can manifest in social systems and institutions ranging from housing to healthcare [21]. Area-level racial bias is measured by aggregating racial bias data from multiple individuals to characterize the ambient social climate, in a geographically defined place, as it pertains to race and racism. Area-level bias may reflect norms and attitudes in a community or may represent a distinct community-level construct and has been shown to predict various domains of population health and health inequities [19,22,23]. For example, states with higher anti-Black bias spend less on disabled Medicaid enrollees [24], and various area-level measures of racial bias have been linked with overall rates of, and racial inequities in: adverse birth outcomes [25], police killings [26], cardiovascular risk factors [27], overall mortality [28], and mortality related to circulatory disease [29,30]. Proposed pathways linking area-level racial bias to health include institutional discrimination [29], disrupted social capital and community cohesion [28], and increased psychosocial stress [23], which can undermine health for *both* racially minoritized *and* White groups [27,28,29,30]. The relationship between area-level racial bias and COVID-19 outcomes have not, to our knowledge, been evaluated. 

The purpose of this study is to estimate the relationship, at the US county level, between non-Hispanic (NH) White people’s implicit and explicit anti-Black racial biases and a number of COVID-19 related outcomes: overall county-level incidence and mortality rates, incidence rates for Blacks and Whites, and gaps in incidence rates between Blacks and Whites. National mortality data disaggregated by race were not available at the time of this writing. We follow previous research [29,30] and focus on county-level biases of Whites as the exposure of interest because COVID-19 outcomes are conceivably driven by racial bias within the most socially and economically advantaged group, due to its greater decision-making power and access to flexible resources. However, previous research has shown that county-level anti-Black bias among Whites predicts poor health outcomes for both Black *and White* populations [29], warranting close examination. Therefore, we hypothesized that higher anti-Black bias would predict higher overall COVID-19 mortality rates, higher incidence rates overall, among Blacks, and among Whites, and, assuming anti-Black bias more strongly impacts health outcomes for Blacks than Whites [25], larger Black–White incidence rate gaps.

## 2. Materials and Methods

### 2.1. Exposure Assessment

Exposure data were drawn from Project Implicit, a large, publicly available source of data on implicit and explicit biases about various social groups and topics [31]. Any person with an Internet connection can take a Project Implicit test at any time, and the results of each test are added to the publicly available dataset of test responses. Some workplaces and schools require participation [31].

Research suggests that previously established discriminatory policies, practices, and norms can create racial inequities in the private and public sectors that may worsen COVID-19 outcomes for Black residents in highly biased areas [7,9]. Thus, we downloaded data for all NH White Implicit Association Test (IAT) respondents of the Black–White race-related tests taken from 01 January 2017 to 31 December 2019 to capture the period preceding the COVID-19 pandemic but after President Trump was formally elected by the Electoral College (i.e., 19 December 2019) because the social climate related to discriminatory norms in the US may have shifted once he became the official President-elect [32].

#### 2.1.1. Implicit Racial Bias

Implicit racial bias was assessed using the Implicit Association Test (IAT), which measures the speed of keyboard associations between images of Black vs. White faces and positive (e.g., “wonderful”) vs. negative (e.g., “disgusting”) words. Faster reaction time matching positive words with White faces and negative words with Black faces indicates cognitive dissonance between Black people and positive emotions. Using the standard scoring algorithm developed by Greenwald et al. [33], implicit bias scores range continuously from −2 to 2, with negative values representing a pro-Black/anti-White bias, positive values representing an anti-Black/pro-White bias, and 0 representing a neutral score. IAT scores were z-score transformed for analysis.

#### 2.1.2. Explicit Racial Bias

Respondents were asked to rank their feelings of warmth/coldness toward Black people and White people on an 11-point Likert scale ranging from 0 “extremely cold” to 10 “extremely warm.” Following previous work [22], we calculated the difference between the White and Black scores (continuous range −10 to 10). Negative values represent warmer feelings toward Black individuals (i.e., explicit pro-Black/anti-White bias), positive values represent warmer feelings toward White individuals (i.e., explicit anti-Black/pro-White bias), and 0 represent a neutral score. Explicit bias was z-score transformed for analysis.

#### 2.1.3. Exclusions

There were n = 2,679,776 implicit and explicit racial bias tests performed from 01 January 2017 to 31 December 2019. We restricted our sample to NH White individuals and excluded those residing outside of the US (territories were excluded). We also excluded those with missing county information to facilitate calculating county-level racial bias scores and merging with COVID-19 data. Following previous research [22,33], we excluded IAT tests where respondents made errors on greater than 30% of trials or had reaction times below 300 milliseconds on more than 10% of trials, thereby omitting bias scores with low accuracy and/or high response latencies. Our final sample size of US-based, NH White respondents with county information and complete and adequate implicit and explicit racial bias data was n = 723,271 across 3017 counties. The characteristics of IAT respondents are presented in Appendix A.

#### 2.1.4. County-Level Averages

We aggregated individual racial bias scores to the county level. The median number of Project Implicit responses per county was 28, ranging from 1 to 13,612 (mean (standard deviation) = 240 (769)). We used a population weighting function which substantially down-weights the relevance of low population counties, and these counties also tend to have fewer Project Implicit tests (Pearson correlation *= r* (3142 counties) = 0.77). Moreover, as we later describe, restricting our analysis to counties with 100 or more Project Implicit responses did not substantially change our results. We thus included as many counties as possible to strengthen power.

### 2.2. Outcome Assessment

Here, “incidence” denotes any ongoing or resolved case of COVID-19, and “mortality” indicates a COVID-19 case that resulted in death. Thus, the county-level per capita incidence rate is the cumulative proportion of all individuals in a given county who have contracted COVID-19 during the study period.

#### 2.2.1. Overall Incidence and Mortality Rates

Overall incidence and death rates from 22 January 2020 to 01 July 2020 were calculated per 10,000 population using data reported by the widely cited Johns Hopkins University Coronavirus Resource Center, an online public-use database tracking daily counts across the US and worldwide [4].

#### 2.2.2. Race-Specific Incidence Rates

Via litigation with the Centers for Disease Control and Prevention, The New York Times (NYT) secured race-disaggregated COVID-19 incidence counts through 28 May 2020 for n = 957 counties [2]. Using these data, NYT calculated Black and White COVID-19 incidence rates per 10,000 population. 

#### 2.2.3. Black–White Incidence Rate Gap

We subtracted the White incidence rate from the Black incidence rate to calculate the difference in incidence rates between the two racial groups, with higher values representing more cases among Black compared to White people.

### 2.3. Covariates

Informed by theory and existing public health literature [1,16,17,34], candidate covariates were selected a priori. Relevant confounders that measure county-level socioeconomic and demographic factors, and political ideology, include median age, percent with a bachelor’s degree, percent Black, percent experiencing household crowding (more than 2 persons per room), population density (persons per-square-mile), and percent living below the federal poverty level. We also include percent voting for Donald Trump in the 2016 presidential election to proxy political and ideological factors affecting social distancing, mask compliance, and testing availability. County-level covariates were assessed using 5-year average estimates from the 2014–2018 American Community Survey (ACS) [35], which were extracted using the acs package in R [36]. Voter data were collected from Politico and accessed via GitHub [37]. Population density was measured using ACS population size divided by land area estimates in ArcGIS [38]. All covariates were assessed continuously. Model fitting details are described in Appendix B.

### 2.4. Study Sample Selection

Our sample in any given model consisted of all US counties for which there was available data for our measures of racial bias and COVID-19 outcomes. As explained above, disaggregated data were available for a smaller subset of counties. Thus, among 3142 US counties (including Washington DC), our samples ranged from 957 (31%) to 2994 counties (95%) depending on the outcome.

### 2.5. Statistical Analysis

Data were cleaned and prepared in R Studio Version 1.2.1335 (R Studio, Boston, MA, USA) and all statistical analysis was performed using STATA 16 (StataCorp LLC, College Station, TX, USA). We report the county-level distributions of NH White implicit and explicit racial biases, covariates, and COVID-19 outcomes. Having established that negative binomial and linear regression yielded similar results for the association between area-level racial bias and incidence and mortality in the overall population (Appendix C), we used multivariable linear regression with robust standard errors to estimate rates and rate differences for ease of interpretation. Following others [25,29,30], our models included counties for which we have data on all measures. We applied analytic weights (proportional to the inverse of the variance of the rate estimate in that county) to counties based on their population sizes to both ensure results are not driven by smaller counties (which are more plentiful), and to account for variation in the precision of our estimates of our exposure and outcome measures (which increases with county population). As described in Appendix B, our model fitting approach was data-driven [39]. Two sets of final models provide estimates of the relationship between each racial bias measure and outcome which are either (1) adjusted for covariates or (2) adjusted for the same set of covariates that were transformed to improve model fit. Detailed descriptions of these transformations are reported in Appendix B.

In all regression models, COVID-19 incidence and mortality rates are per 10,000 people. All regression coefficients (and 95% confidence intervals (CI)) have been standardized such that they indicate the shift in a given rate outcome associated with a one standard deviation increase in the predictor (after adjustment for other covariates). This format allows comparability across racial bias measures. Only main associations are reported: final models adjusted for covariates are shown in Appendix D.

### 2.6. Sensitivity Analyses

As described in Appendix E, we performed the following sensitivity analyses: (1) restricting to first-time Project Implicit respondents since racial bias scores among repeated test-takers may regress toward the mean [40], (2) constructing our exposures by aggregating across Whites who specifically identified as non-Hispanic (rather than across Whites who did not identify as Hispanic), (3) restricting to counties with at least 20 (and then again with at least 100) Project Implicit respondents to avoid unstable estimates [25], and (4) restricting all analyses to the 957 counties for which we have NYT race-specific data.

## 3. Results

### 3.1. Sample Characteristics

Table 1 shows the distribution of county-level NH White racial bias scores, COVID-19 rates, and covariate values. Across 2994 counties, NH Whites’ implicit and explicit (hereafter implicit and explicit) racial bias had mean (standard deviation (SD)) scores of 0.38 (0.14) and 0.40 (0.62), respectively. These were very similar to average bias levels in the 957-county set (0.38 (0.14) and 0.40 (0.38)). Incidence and mortality rates are reported per 10,000 people. Across 2994 counties, the average COVID-19 mortality rate was 1.72 per 10,000 (3.33) and incidence rate was 52.1 per 10,000 (77.2) for the overall population. Across the 957-county set, these rates were 3.15 (4.42) and 82.05 (86.57). Average confounder values for the two county sets were generally similar, with the exception of population density and percent Black, which were higher in the smaller subset of counties. Across the 957 counties, the average incidence rate among Whites (16.5 (27.5)) was lower than among Blacks (35.6 (92.5)), for an average Black–White gap in incidence rate of 19.1 (78.8). 

### 3.2. Multivariable Regression

In models adjusted for covariates, a standard deviation higher average implicit bias was associated with: a higher overall COVID-19 incidence rate (b = 12.11 (6.96, 17.25)), higher overall COVID-19 mortality rate (b = 0.9 (0.54, 1.25)), higher incidence rate among Whites (b = 4.20 (1.65, 6.75)), higher incidence rate among Blacks (b = 10.25 (5.00, 15.51)), and larger Black–White incidence rate gap (b = 6.05 (2.61, 9.50)).

Higher average explicit bias was also associated with worse COVID-19 outcomes in adjusted models: a higher COVID-19 incidence rate (b = 11.24 (6.35, 16.13)), higher overall mortality rate (b = 0.65 (0.32, 0.98)), higher incidence rate among Whites (b = 4.27 (1.56, 6.98)), higher incidence rate among Blacks (b = 13.1 (8.45, 17.75)), and larger Black–White incidence rate gap (b = 8.83 (5.42, 12.24)).

The above pattern of findings largely holds in more flexible models incorporating confounder controls, variable transformations, and interactions, with one exception: after adjusting for transformed and interacted confounders, 95% confidence intervals for implicit bias as a predictor of the Black–White incidence rate gap encompass the null. All regression estimates are presented in Table 2.

### 3.3. Sensitivity Analyses

Models performed similarly to our main models when we restricted to first-time Project Implicit test-takers, to counties with at least 20 Project Implicit tests, to counties with at least 100 tests, and to the 957 counties in the NYT data, and when we limited our Project Implicit sample to White individuals who explicitly identified as non-Hispanic (rather than to those who did not identify as Hispanic). The notable exception was that after restricting our analysis to counties with at least 100 Project Implicit tests, implicit bias became a statistically significant predictor of the Black–White COVID incidence rate gap in models adjusting for transformed and interacted confounders.

## 4. Discussion

Our ecologic study is the first to report associations of county-level NH Whites’ implicit and explicit racial bias with COVID-19 incidence and mortality rates in the United States. This finding aligns with previous evidence linking area-level racial bias with rates of disease and mortality, and is among the first to explore this relationship with an infectious rather than chronic disease. [25,29]. We found that county-level average racial bias among NH Whites was positively associated with COVID-19 incidence rates among both Black and White residents, consistent with prior work suggesting that area-level racial bias may be harmful for everyone’s health [28,29,41]. Finally, higher levels of explicit (but not implicit) anti-Black bias were associated with greater Black–White gaps in COVID-19 incidence rates after accounting for other predictors. This finding is consistent with prior work showing area-level explicit racial bias to be a stronger predictor of racial inequities in health outcomes when compared with implicit bias [25,29]. Below, we posit several potential pathways linking county-level racial bias of NH Whites with COVID-19 incidence, mortality, and inequities to guide future research and inform targeted interventions.

Whites’ anti-Black racial bias may flow through political and social inequalities that can lead to Black–White inequities in COVID-19 incidence and disease severity. For instance, racially biased decision making by private and public sector arbiters in high bias counties may result in disparate opportunities for members of different racial groups. Research has found, for example, that Whites’ racial bias is linked to Black-White income inequality [42]. Biased decision making could also contribute to racial inequities in COVID-19 outcomes through differences in access to protective resources such as private versus public transportation options, physical distancing versus crowding within various spaces (e.g., homes, neighborhoods, workplaces), access to quality healthcare, and other structural factors. In addition, Black people are over-represented among lower wage workers deemed as “essential” in the workforce (e.g., caregivers, bus drivers, cashiers), and thereby maintain higher risk of COVID-19 exposure compared to White people who are more likely to be able to work from home [43,44]. Further, ongoing inequities such as residential segregation and differential access to quality housing, healthcare, higher education, and higher wages have long contributed to Black–White inequities in a range of illnesses including cardiovascular disease and risk of mortality [29,30,45], all of which are associated with increased risk of COVID-19 infection and worse disease severity [46]. Finally, in counties with higher NH White racial bias, Black people report decreased access to healthcare services [29]. Future work may consider evaluating whether structural inequities, such as income inequality and healthcare access, partially mediate the association between area-level racial bias and racial inequities in COVID-19 incidence and mortality.

Community-level social capital—defined as an arrangement of resources that are accessed by community members [47]—is another plausible mechanism linking racial bias with county-level COVID-19 incidence and mortality. A recent study showed that counties with higher social capital operating as civic norms that foster cooperation, collective action, and community trust had increased social distancing [48]. Community-level social capital has also been shown to mediate associations between community-level racial prejudice and mortality among Black and White residents [28], which may help explain, in part, our finding of higher incidence among Whites. Indicators of social capital include interpersonal trust, civic and political engagement, and access to services and resources [47]. For example, socially cohesive communities are more successful at collective lobbying to preserve local services and amenities [49,50], benefiting members of advantaged and disadvantaged racial groups. It is conceivable that residents living in counties with higher anti-Black bias are less trusting of community members, leading to the erosion of social capital, which is harmful for both Black and White residents. Support for shared resources and programs that benefit the population as a whole may be especially important in a pandemic for the poor, homeless, institutionalized elderly, and other low-status groups in biased areas, regardless of race. Lower social capital could also disrupt trust and reciprocity between community members, possibly reducing the motivation to practice collective action to protect each other from COVID-19 by wearing a mask, physical distancing, or sharing resources and information. Social capital may be improved by public policies aimed to reduce racial residential, educational, and occupational segregation, thereby fostering meaningful intergroup contact, which has been shown to reduce prejudice and improve in-group connections, cross-group empathy, and community trust [51,52,53]. Examining changes in social capital as a moderator or mediator of the association between county-level racial bias and COVID-19 outcomes is an important area for future research [28].

A final plausible mechanism linking county-level racial bias with COVID-19 outcomes and inequities is greater overall exposure to psychosocial stress. Counties where NH White individuals harbor greater levels of anti-Black racial bias may be characterized by greater racial tension and hostility, leading to stressful interracial interactions which can trigger biopsychosocial stress-response processes [54,55]. These processes, in turn, may decrease immunity [56], increase generalized susceptibility [57] to infectious agents [6], and lead to physical and mental exhaustion which impairs adherence to health-preserving behavioral changes, such as mask-wearing. While community tension and hostility may create stress for everyone, the effects may be disproportionately harmful for Black residents. For example, some have suggested that Black individuals living in areas with higher levels of racial bias may encounter more interpersonal and institutional racial discrimination [23,25], a toxic psychosocial stressor associated with myriad adverse health outcomes [20]. They may also be more likely to experience uniquely stressful life events (such as racism-related job losses or police encounters) [54]. Moreover, living in counties with higher levels of anti-Black bias may cause stress from collective or vicarious racism experiences, even in the absence of direct interpersonal racism encounters [54]. Finally, chronic contextual stress (stress resulting from the socio-political climate and inequitable social structure) [54] may be more salient in counties with more racial bias. Indeed, previous work has linked community-level racial bias with stress-related outcomes among racially minoritized groups, including cardiovascular risk factors and adverse birth outcomes [25,27,29]. Future research should examine these potential mediating pathways linking area-level bias to COVID-19 and other adverse health outcomes.

There are important methodological considerations. This cross-sectional, ecologic study leveraged several large, publicly available national datasets. The design was not intended to establish causality, but rather to determine whether, for whom, and to what extent, county-level racial bias is associated with COVID-19 outcomes. We utilize Project Implicit data to ascertain racial bias in part because implicit bias data do not rely on self-report, thereby circumventing the self-censorship inherent in many measures of racial attitudes. Importantly, county-level racial bias scores are only representative of those who chose to take the IAT, which limits generalizability. However, previous work showed high construct validity of county-level IAT scores in comparison to several nationally representative sources of data on racial attitudes [22]. 

Temporal factors and data limitations present a threat to external validity. Outcome data are comprised of county-level cases and deaths that occurred relatively early in the pandemic, during which time outbreaks were clustered in locations with high population densities (e.g., New York City, nursing homes, meatpacking plants) and may not be generalizable to more rural or sparsely populated areas. Further, race-disaggregated data excludes the surge of COVID-19 cases in the Sun Belt during the summer and in the Midwest during the fall; hence, counties in the Northeast are likely overrepresented. Future research with more national data and a longer time frame will bolster our understanding of these associations and whether they change over the course of the pandemic. Critically, disaggregated data on morality rates were not available, and disaggregated data on incidence rates were only available for 957 counties. As noted in Table 1, the 957 counties with race-disaggregated data had higher overall COVID-19 incidence and mortality rates, higher percentages of Black individuals, higher populations, and higher population densities compared to counties for which race-disaggregated data were not available. Additionally, the disaggregated cases occurred around a time when 83% of the population resided in US counties with high COVID-19 prevalence [58]. Given the characteristics of these 957 counties, our disaggregated results may be most appropriately generalized to more populous and diverse counties.

As noted, our goal was to evaluate the association between racial bias and COVID-19 outcomes. Internal validity in this regard is strengthened by our theory- and data-driven approaches to address confounding. Still, unmeasured confounding certainly remains. For example, we adjust for percent voting for Trump in 2016 in part to proxy political and ideological factors that may contribute COVID-19 spread and detection. However, this indicator is imperfect and there likely remains confounding via unmeasured socio-political pathways through which NH whites’ county-level racial bias may influence COVID-19 outcomes. In addition, variation in *R^2^* values suggests that some variables in our collective set of covariates predict more of the variance than others. For instance, our variable set predicts more variance in overall mortality than in overall incidence. This suggests that some variables that are not included in the model are particularly important predictors of incidence but less so for predicting mortality.

## 5. Conclusions

Greater county-level anti-Black bias among non-Hispanic Whites is associated with higher overall COVID-19 mortality and incidence rates, higher incidence rates for Whites and Blacks, and higher White-Black incidence rate gaps after accounting for other county-level predictors. Racial bias helps explain not only where COVID-19 is most negatively impacting individuals of all races, but also where the disease is disproportionately impacting Black individuals. This has critical implications for future research and present policy. Future studies should examine mediators and modifiers of the association between anti-Black bias and COVID-19 outcomes, especially mechanisms amenable to short-term policy responses. Public policies promoting meaningful community-level intergroup contact may help mitigate the effects of racial bias through increased cross-group trust and empathy, and social capital. Given the incomplete nature of available data on this critical issue, researchers and policymakers should prioritize the development of a national registry of COVID-19 patients including demographic characteristics. Finally, while there are certainly many direct steps counties should consider to drive-down COVID-19 inequities, reducing area-level racial bias may be essential to ensuring successful efforts.

## Figures and Tables

**Table 1:** US county-level distribution of racial bias, COVID-19 incidence and mortality rates, and socioeconomic, demographic, and political variables for the samples of 2994 counties (in models assessing overall rates using Johns Hopkins data) and 957 counties (in models assessing race-disaggregated rates using The New York Times (NYT) data).

Variable	n	Mean	SD	Min.	Max.
Implicit Bias					
Unstandardized	2994	0.38	0.14	−1.16	1.42
	957	0.38	0.08	−0.21	0.84
Standardized	2994	0.00	1.00	−10.86	7.24
	957	0.00	1.00	−7.30	5.62
Explicit Bias					
Unstandardized	2994	0.40	0.62	−10.00	8.00
	957	0.40	0.38	−1.75	3.33
Standardized	2994	0.00	1.00	−16.86	12.33
	957	0.00	1.00	−5.66	7.73
Covariates					
Median Age	2994	41.25	5.27	21.70	67.00
	957	40.12	4.58	24.60	67.00
Percent with Bachelor’s Degree	2994	21.70	9.48	5.38	78.53
	957	24.77	10.77	8.18	74.56
Percent Black	2994	9.08	14.16	0.00	82.61
	957	13.87	17.27	0.01	82.61
Percent in Poverty	2994	15.50	6.19	2.30	55.10
	957	15.23	6.13	3.46	41.75
Percent in Crowded Housing	2994	2.30	1.74	0.00	15.46
	957	2.22	1.47	0.00	13.06
Percent Voting Trump (2016)	2994	63.55	15.21	4.12	91.86
	957	56.30	14.46	9.59	89.96
Population Density	2994	283.03	1834.28	0.24	71,509.98
	957	540.64	3055.53	1.95	71,509.98
Population Size	2994	111,296	371,533	463	10,000,000
	957	195,433	448,658	3838	8,336,817
Mortality Rate (per 10,000)					
Overall Mortality Rate, 7/1/20	2994	1.72	3.33	0.00	37.84
	957	3.15	4.42	0.00	37.84
Incidence Rate (per 10,000)					
Overall Incidence Rate, 7/1/20	2994	52.12	77.21	0.00	1319.57
	957	82.05	86.57	0.00	864.93
White Incidence Rate, 5/28/20	957	16.48	27.51	0.00	455.00
Black Incidence Rate, 5/28/20	957	35.59	92.45	0.00	1181.00
Black–White Incidence Rate Gap, 5/28/20	957	19.11	78.80	−204.00	1069.00

SD = standard deviation. While there were 3142 counties for which we had data on any given variable, our adjusted models had either 2994 counties (for overall rate models) or 957 counties (for race-disaggregated rate models) as these were the counties which had information on all model-relevant variables. Above, we report averages for the 2994 and 957 county sets to demonstrate similarities and differences between county sets. Unstandardized exposure summary statistics provide a sense of aggregate racial bias central tendency, which trend slightly above 0 (indicating general pro-White bias). Standardized exposure measures provide ease of comparability against regression coefficients, which are x-standardized and reported later.

**Table 2:** Associations of implicit and explicit racial bias with COVID-19 rates per 10,000 individuals in the county using standardized coefficients: adjusted for confounders (Adjusted) or adjusted for confounders with relevant transformations and interactions (Adjusted and Transformed).

Anti-Black Racial Bias		Overall Mortality Rate (95% CI)	Overall Incidence Rate (95% CI)	White Incidence Rate (95% CI)	Black Incidence Rate (95% CI)	Black–White Incidence Rate Gap (95% CI)
	N	A: 2,994/ AT: 2,933	A: 2,994/ AT: 2,933	957	957	957
Implicit Bias	Adjusted	0.9 *** (0.54, 1.25)	12.11 *** (6.96, 17.25)	4.20 ** (1.65, 6.75)	10.25 *** (5.00, 15.51)	6.05 ** (2.61, 9.50)
	*R^2^*	0.50	0.24	0.54	0.30	0.17
	Adjusted and Transformed	0.65 *** (0.39, 0.91)	8.42 *** (4.64, 12.20)	4.89 *** (2.19, 7.59)	7.54 ** (2.10, 12.97)	2.65 (−0.93, 6.23)
	*R^2^*	0.60	0.38	0.62	0.32	0.18
Explicit Bias	Adjusted	0.65 *** (0.32, 0.98)	11.24 *** (6.35, 16.13)	4.27 ** (1.56, 6.98)	13.1 *** (8.45, 17.75)	8.83 *** (5.42, 12.24)
	*R^2^*	0.49	0.24	0.54	0.31	0.17
	Adjusted and Transformed	0.49 *** (0.27, 0.70)	8.83 *** (5.32, 12.35)	4.18 ** (1.75, 6.61)	10.29 *** (5.43, 15.15)	6.11 ** (2.68, 9.54)
	*R^2^*	0.59	0.38	0.62	0.32	0.19

CI = confidence interval. Models estimate COVID-19 outcome rates per 10,000 individuals (and associated 95% confidence intervals). Models involving implicit and explicit bias were run separately, totaling 20 models. Coefficients are standardized on x such that each indicates the shift in y (unstandardized) associated with a one standard deviation increase in x. “Adjusted” (A) models are adjusted for median age, percent with bachelor’s degree, percent Black, percent in poverty, percent in crowded housing, population density, and percent voting Trump. “Adjusted and Transformed” (AT) models are adjusted for median age, percent with bachelor’s degree, percent with a bachelor’s degree-squared, the log of percent Black, percent in poverty, percent in crowded housing, population density, percent voting Trump, percent experiencing crowding X population density, percent with a bachelor’s degree X the log of percent Black, percent with a bachelor’s degree squared X the log of percent Black, and median age X percent voting for Trump. AT models on overall mortality and incidence rates are based on n = 2933 counties as 61 counties from the larger set of 2994 counties had percentage Black values equal to 0, thus the natural log could not be defined in those cases, resulting in the omission of these counties. Inclusion, with imputed, extremely low values for percent Black 0.007 (the lowest non-zero value occurring in our data), did not change results. To avoid imputation, we present results based on the 2933 counties for which log values could be calculated. ** *p* < 0.01, *** *p* < 0.001

## Appendix A. Characteristics of IAT Respondents Used to Construct County-Level Racial Bias Scores

**Table A1:** Characteristics of IAT test-takers aggregated to create county average IAT scores, 2017–2019 (n = 723,256).

**Characteristic**	**n (%)**
Gender identity	
Woman	451,908 (62.5)
Man	262,992 (36.4)
Nonbinary	7939 (1.1)
NA	417 (0.1)
Age (mean (SD))	29.7 (13.9)
Educational attainment	
<high school	97,418 (13.5)
high school graduate	283,493 (39.2)
bachelor’s degree	340,940 (47.1)
Missing	1405 (0.2)
Number previous tests	
0	477,404 (66.0)
1	143,625 (19.9)
2	52,608 (7.3)
3–5	39,000 (5.4)
6 +	9861 (1.4)
Missing	758 (0.1)
Implicit racial bias (mean (SD))	0.36 (0.41)
Explicit racial bias (mean (SD))	0.24 (1.54)

Project Implicit sample restricted to NHW, US-based respondents with complete and adequate test data and county data.

## Appendix B. Confounder Selection and Transformations

### Appendix B.1. Confounder Selection

As mentioned in our manuscript, candidate confounders were chosen a priori and included county-level median age, percent with a bachelor’s degree, percent Black, percent experiencing household crowding, population density, percent living below the federal poverty level, percent of voting for Donald Trump in the 2016 presidential election, and the Index of Concentration at the Extremes (ICE)—a measure of racialized economic segregation (high-income White households minus low-income Black households, ranging from −1 to 1) [59]. Covariates are coded and assessed continuously.

Model specifications were evaluated using data-driven approaches [39]. Possible confounders were assessed using several regressions: (1) direct covariate–outcome associations and (2) main exposure–outcome association adjusted for one covariate at a time. Pearson correlations were estimated among all covariates. Principal components analysis evaluated which covariates explained most of the variance-covariance within the data structure. Multicollinearity between covariates was assessed using variance inflation factors (VIF). Final models were appraised for best fit using nested modeling and Akaike and Bayesian information criteria (AIC and BIC).

Median age, percent bachelor’s degree, percent Black, population density, and percent voting for Trump were directly associated with COVID-19 incidence and confounded main exposure–outcome association when adjusted for one single covariate at a time (*p* < 0.05). The same was found for COVID-19 death rates with the exception of implicit bias adjusted for median age. Strong inverse correlations were found for ICE with percent Black (*r* = −0.74) and percent persons in poverty (*r* = −0.76). Almost 100% of the first principal component explained the variance-covariance, with the strongest contributors being percent poverty, percent Black, and ICE (|0.46–0.55|). We ultimately omitted ICE, but retained percent Black and percent poverty, from future model-building evaluations for two reasons. First, ICE is comprised of percent Black and percent poverty. Secondly, ICE was not directly associated with COVID-19 incidence or death rates. No multicollinearity was detected in regression models including all remaining covariates (VIF < 4).

Nested model building favored the inclusion median age, percent bachelor’s degree, percent Black, population density, and percent voting for Trump. AIC and BIC values showed that the best fit models included the same covariates favored in the nested models, in addition to percent poverty and percent crowding. Because AIC and BIC values suggest that including percent poverty and percent crowding improve model fit, and because poverty and household crowding are strong predictors of COVID-19 deaths [1], percent poverty and percent crowding were included in our analysis. Hence, our final models were adjusted for median age, percent bachelor’s degree, percent Black population, persons in poverty, percent household crowding, percent population density, and percent voting for Trump in 2016.

### Appendix B.2. Confounder Transformations and Interactions

As noted in the body of the manuscript, we attempted to obtain a less biased estimate of the relationship between our bias measures and COVID-19 outcome measures by adjusting for seven covariates: median age, percent with a bachelor’s degree, percent Black, percent in poverty, percent experiencing crowding (meaning more than two per room), population density, and percent who voted for President Trump in 2016.

The use of linear regression to estimate the relationship between any two variables assumes linear relationships between all predictors and outcome and the inclusion of relevant interaction terms. To reduce bias in our estimate of the relationship between bias and outcomes, we thus sought to discern which, if any, confounders to transform and to interact with one another. We first reviewed the shape of the relationship between each confounder and each outcome using locally weighted regressions via the “loess” command in STATA 16. We then created appropriate transformed versions of each confounder which exhibited a non-linear relationship with our outcomes (creating square terms for parabolic relationship, creating log terms for diminishing return shaped relationships, etc.). Next, we employed likelihood ratio tests to determine which of transformations performed better than their untransformed analogues, and discerned that log percent Black and percent with a Bachelor degree with a square term performed better than their analogues, with χ^2^ likelihood ratio values of 874.14 (*p* < 0.0001) and 49.89 (*p* < 0.0001) respectively, on their ability to predict the overall COVID-19 rate.

We next discerned which, if any, 1 × 1 interactions to include in our models. We vetted each potential interaction, then included the set of three 1 × 1 interactions which did the most to improve our model fit. These were interaction terms on percent experiencing crowding with population density, percent with a bachelor’s degree (and its square term) with the log of percent Black, and median age with percent voting for Trump in 2016.

Including these additional terms improved model fit from an average R^2^ value in adjusted models of 0.36 to an average R^2^ value in adjusted and transformed models of 0.43, and markedly improved the fit in some models by a factor of as much as 1.6.

**Table A2:** *R^2^* values in adjusted versus adjusted and transformed models.

Predictor	Outcome	Adjusted Models	Adjusted and Transformed Models
Implicit bias	Overall incidence rate	0.24	0.38
	White incidence rate	0.54	0.62
	Black incidence rate	0.30	0.32
	Black–White rate gap	0.17	0.18
	Overall mortality rate	0.50	0.60
Explicit Bias	Overall incidence rate	0.23	0.38
	White incidence rate	0.54	0.62
	Black incidence rate	0.50	0.60
	Black–White rate gap	0.17	0.19
	Overall mortality rate	0.49	0.59
Average		0.36	0.43

## Appendix C. Negative Binomial Regression Results

**Table A3:** Comparing linear regression and negative binomial regression models: COVID-19 incidence and mortality rates associated with a one unit increase in county-level implicit and explicit bias (adjusted for other predictors), per 10,000 individuals in the county (n = 2994).

	Incidence Rate		Mortality Rate	
	Linear	Negative Binomial	Linear	Negative Binomial
Implicit Bias	215.5 *** (123.9, 307.1)	213.6 *** (144.1, 283.0)	16.0 *** (9.6, 22.3)	18.9 *** (11.6, 26.3)
*R^2^*	0.24	0.01 ^Ɨ^	0.50	0.03 ^Ɨ^
Explicit Bias	44.2 *** (24.9, 63.4)	51.7 *** (33.4, 70.1)	2.6 *** (1.3, 3.8)	4.1 *** (2.1, 6.2)
*R^2^*	0.23	0.01 ^Ɨ^	0.49	0.03 ^Ɨ^

Models estimate COVID-19 outcome rates per 10,000 individuals (and associated 95% confidence intervals). ^Ɨ^ Pseudo R^2^. *** *p* < 0.001

## Appendix D. Regression Model Results

**Table A4:** Predicted shift in COVID-19 rates associated with a one standard deviation increase in county-level implicit bias (adjusted for other predictors), per 10,000 individuals in the county.

Variable	Overall Mortality Rate	Overall Incidence Rate	White Incidence Rate	Black Incidence Rate	Black–White Incidence Rate Gap
n	2994	2994	957	957	957
Implicit Bias	0.9 *** (0.54, 1.25)	12.11 *** (6.96, 17.25)	4.20 ** (1.65, 6.75)	10.25 *** (5.00, 15.51)	6.05 ** (2.61, 9.50)
Median Age	0.48 ** (0.17, 0.78)	−1.17 (−5.66, 3.32)	4.07 ** (1.54, 6.60)	1.63 (−4.15, 7.40)	−2.44 (−6.59, 1.71)
Percent with Bachelor’s Degree	0.41 (−0.25, 1.07)	5.87 (−4.13, 15.87)	−4.99 (−10.55, 0.57)	−9.98 (−22.22, 2.27)	−4.99 (−13.71, 3.74)
Percent Black	0.08 (−0.48, 0.64)	9.68 * (1.91, 17.45)	1.49 (−1.68, 4.66)	−2.53 (−9.84, 4.77)	−4.02 (−8.94, 0.90)
Percent in Poverty	−0.1 (−0.56, 0.36)	−1.96 (−9.39, 5.48)	−6.77 ** (−10.79, −2.74)	−16.07 *** (−23.68, −8.45)	−9.3 *** (−14.39, −4.21)
Percent in Crowded Housing	−1.02 * (−1.83, -0.21)	−0.1 (−11.60, 11.40)	20.18 *** (11.68, 28.68)	12.24 * (2.90, 21.59)	−7.94 ** (−13.07, −2.80)
Population Density	3.31 *** (2.38, 4.25)	19.12 ** (6.20, 32.05)	3.63 (−3.89, 11.15)	15.22 ** (4.50, 25.94)	11.59 *** (5.70, 17.49)
Percent Voting Trump	−1.47 *** (−2.16, -0.79)	−13.95 ** (−24.11, −3.80)	−12.54 *** (−17.70, −7.38)	−36.38 *** (−47.96, −24.79)	−23.83 *** (−32.08, −15.59)
*R^2^*	0.50	0.24	0.54	0.30	0.17

Models estimate COVID-19 outcome rates per 10,000 individuals (and associated 95% confidence intervals). Coefficients are standardized on x such that each indicates the shift in y (unstandardized) associated with a one standard deviation increase in x. * *p* < 0.05, ** *p* < 0.01, *** *p* < 0.001

**Table A5:** Predicted shift in COVID-19 rates associated with a one standard deviation increase in explicit bias (adjusted for other predictors), per 10,000 individuals in the county.

Variable	Overall Mortality Rate	Overall Incidence Rate	White Incidence Rate	Black Incidence Rate	Black–White Incidence Rate Gap
n	2994	2994	957	957	957
Explicit Bias	0.65 *** (0.32, 0.98)	11.24 *** (6.35, 16.13)	4.27 ** (1.56, 6.98)	13.1 *** (8.45, 17.75)	8.83 *** (5.42, 12.24)
Median Age	0.56 *** (0.26, 0.86)	0.05 (−4.49, 4.59)	4.92 *** (2.34, 7.50)	3.73 (−1.90, 9.35)	−1.19 (−5.22, 2.83)
Percent with Bachelor’s Degree	0.39 (−0.28, 1.06)	5.64 (−4.44, 15.72)	−4.78 (−10.23, 0.67)	−9.34 (−21.35, 2.67)	−4.56 (−13.17, 4.04)
Percent Black	0.15 (−0.43, 0.73)	10.28 * (2.39, 18.16)	1.76 (−1.42, 4.94)	−2.57 (−9.92, 4.78)	−4.33 (−9.29, 0.64)
Percent in Poverty	−0.11 (−0.59, 0.36)	−1.89 (−9.47, 5.69)	−6.65 ** (−10.64, −2.67)	−15.3 *** (−22.67, −7.93)	−8.65 ** (−13.62, −3.68)
Percent in Crowded Housing	−0.94 * (−1.74, −0.15)	1.19 (−10.11, 12.48)	20.72 *** (12.13, 29.30)	14.12 ** (5.06, 23.17)	−6.6 * (−11.93, −1.27)
Population Density	3.32 *** (2.35, 4.29)	19.25 ** (5.96, 32.54)	3.58 (−3.80, 10.95)	14.9 ** (4.43, 25.38)	11.33 *** (5.45, 17.20)
Percent Voting Trump	−1.43 *** (−2.13, −0.72)	−14.52 ** (−25.09, −3.94)	−12.88 *** (−18.44, −7.31)	−38.68 *** (−50.12, −27.23)	−25.8 *** (−33.88, −17.72)
*R^2^*	0.49	0.24	0.54	0.31	0.17

Models estimate COVID-19 outcome rates per 10,000 individuals (and associated 95% confidence intervals). Coefficients are standardized on x such that each indicates the shift in y (unstandardized) associated with a one standard deviation increase in x. * *p* < 0.05, ** *p* < 0.01, *** *p* < 0.001

## Appendix E. Sensitivity Results

### Appendix E.1. Complete Sensitivity Analysis

**Table A6:** Complete sensitivity analyses.

Outcome	Model Type	Implicit Bias		Explicit Bias	
		Adjusted	Adjusted and Transformed	Adjusted	Adjusted and Transformed
**Overall Mortality Rate**	Main model	15.95 *** (9.57, 22.34)	11.68 *** (7.08, 16.28)	2.56 *** (1.28, 3.85)	1.93 *** (1.07, 2.79)
	First time test-takers	12.69 *** (7.72, 17.65)	8.92 *** (5.54, 12.30)	2.02 *** (1.03, 3.02)	1.48 *** (0.82, 2.13)
	Explicitly NHW	14.38 *** (8.40, 20.35)	10.56 *** (6.27, 14.85)	2.23 *** (1.03, 3.44)	1.62 *** (0.83, 2.41)
	> = 20 PI tests	33.94 *** (21.51, 46.36)	25.26 *** (15.80, 34.71)	5.06 *** (2.50, 7.62)	3.76 *** (2.01, 5.51)
	> = 100 PI tests	59.87 *** (39.39, 80.35)	47.23 *** (29.82, 64.63)	8.02 *** (3.76, 12.28)	5.93 *** (2.90, 8.96)
	NYT data only	36.84 *** (20.86, 52.82)	27.05 *** (15.05, 39.04)	5.33 ** (2.11, 8.54)	4.17 *** (2.01, 6.33)
**Overall Incidence Rate**	Main model	215.52 *** (123.92, 307.12)	151.34 *** (83.37, 219.31)	44.16 ** (24.95, 63.38)	34.95 *** (21.04, 48.85)
	First time test-takers	169.32 *** (96.01, 242.62)	111.76 *** (59.18, 164.35)	34.09 *** (18.85, 49.34)	26.02 *** (14.95, 37.09)
	Explicitly NHW	197.94 *** (112.45, 283.44)	140.70 *** (77.46, 203.94)	37.70 *** (19.86, 55.54)	28.61 *** (15.92, 41.31)
	> = 20 PI tests	464.22 *** (286.67, 641.77)	332.24 *** (194.55, 469.94)	78.48 *** (41.08, 115.87)	58.35 *** (31.32, 85.38)
	> = 100 PI tests	820.77 *** (545.25, 1096.28)	632.88 *** (404.57, 861.19)	114.86 *** (53.76, 175.95)	83.08 *** (37.94, 128.22)
	NYT data only	554.86 *** (325.78, 783.93)	386.04 *** (229.00, 543.09)	93.58 *** (46.12, 141.05)	74.96 *** (46.76, 103.16)
**White Incidence Rate**	Main model	91.78 ** (35.97, 147.58)	106.82 *** (47.79, 165.85)	20.13 ** (7.33, 32.93)	19.71 *** (8.23, 31.18)
	First time test-takers	74.95 ** (30.07, 119.84)	86.11 *** (39.03, 133.18)	18.31 ** (7.33, 29.29)	16.94 *** (7.49, 26.40)
	Explicitly NHW	84.57 ** (30.19, 138.95)	95.86 *** (39.61, 152.12)	17.43 ** (5.16, 29.70)	16.93 ** (5.90, 27.96)
	> = 20 PI tests	142.88 *** (67.12, 218.65)	169.82 *** (91.62, 248.02)	30.08 ** (12.09, 48.07)	29.06 *** (13.21, 44.92)
	> = 100 PI tests	204.64 *** (92.49, 316.79)	249.98 *** (139.82, 360.14)	40.88 ** (15.77, 65.99)	38.19 *** (16.46, 59.91)
**Black Incidence Rate**	Main model	224.04 *** (109.03, 339.04)	164.71 ** (45.80, 283.62)	61.79 *** (39.81, 83.77)	48.54 *** (25.59, 71.48)
	First time test-takers	168.33 *** (68.96, 267.70)	121.74 * (20.03, 223.44)	50.83 *** (33.01, 68.64)	39.32 *** (20.96, 57.69)
	Explicitly NHW	196.46 *** (87.85, 305.06)	140.38 * (27.79, 252.97)	56.79 *** (34.75, 78.82)	45.12 *** (22.30, 67.94)
	> = 20 PI tests	336.54 *** (186.21, 486.86)	257.76 ** (97.89, 417.63)	81.19 *** (52.77, 109.60)	65.51 *** (35.77, 95.25)
	> = 100 PI tests	476.09 *** (260.10, 692.08)	410.74 *** (191.00, 630.48)	108.91 *** (68.56, 149.26)	89.05 *** (47.33, 130.76)
**Black–White Incidence Rate Gap**	Main model	132.26 *** (56.88, 207.64)	57.89 (−20.40, 136.18)	41.66 *** (25.55, 57.78)	28.83 *** (12.63, 45.03)
	First time test-takers	93.38 ** (23.70, 163.05)	35.63 (−36.55, 107.81)	32.51 *** (20.08, 44.95)	22.38 *** (9.83, 34.93)
	Explicitly NHW	111.88 ** (41.26, 182.51)	44.52 (−28.82, 117.85)	39.36 *** (23.09, 55.63)	28.19 *** (11.90, 44.49)
	> = 20 PI tests	193.65 *** (96.59, 290.71)	87.94 (−19.01, 194.89)	51.11 *** (30.57, 71.65)	36.45 *** (15.62, 57.27)
	> = 100 PI tests	271.45 *** (135.03, 407.87)	160.76 * (14.91, 306.60)	68.03 *** (38.21, 97.85)	50.86 *** (21.06, 80.66)

NHW = non-Hispanic White; NYT = The New York Times. Estimates correspond to rate differences (and 95% confidence intervals). All outcomes are calculated per 10,000 individuals. * *p* < 0.05, ** *p* < 0.01, *** *p* < 0.001

### Appendix E.2. First-Time IAT Test-Takers

Project Implicit respondents report the number of times they have previously taken the IAT. For our primary analysis, we retained all racial bias tests, regardless of whether the respondent had previously taken a test. This decision was motivated by our goal of capturing the current social context in each county over a specified time period (2017–2019). Therefore, if a respondent took the IAT in 2009 and again in 2018, we would want to include their score in our measure of 2017–2019 county-level racial bias. Similarly, we would want to retain a respondent’s racial bias data even if they previously took the IAT in a different county. Because the dataset does not indicate when or where a previous test was taken, we could not selectively include or exclude previous tests. However, as a sensitivity analysis, we excluded those who had taken the test previously (n= 245,101) or who did not report on number of previous tests (n = 758), restricting the sample to first-time IAT respondents (66.6%) [40]. Basic demographics and racial bias scores between first-time and repeat test-takers were very similar, with slightly higher racial bias scores among first-time test-takers compared to those who have previously taken the IAT.

**Table A7:** Characteristics of IAT test-takers by previous tests, 2017–2019.

Characteristic	Primary Sample: All Respondents (n = 723,271)	Sensitivity Sample: First Time Respondents (n = 477,404)
Gender identity	n (%)	n (%)
Woman	451,908 (62.5)	286,744 (60.1)
Man	262,992 (36.4)	185,994 (39.0)
Nonbinary	7939 (1.1)	4395 (0.9)
NA	417 (0.1)	271 (0.1)
Age (mean (SD))	29.7 (13.9)	29.4 (14.1)
Educational attainment		
<high school	97,418 (13.5)	75,499 (15.8)
high school graduate	283,493 (39.2)	190,310 (39.9)
bachelor’s degree	340,940 (47.1)	210,677 (44.1)
Missing	1405 (0.2)	918 (0.2)
Number previous tests		
0	477,404 (66.0)	477,404 (100)
1	143,625 (19.9)	0
2	52,608 (7.3)	0
3–5	39,000 (5.4)	0
6 +	9861 (1.4)	0
Missing	758 (0.1)	0
Implicit racial bias (mean (SD))	0.36 (0.41)	0.39 (0.40)
Explicit racial bias (mean (SD))	0.24 (1.54)	0.29 (1.55)

Primary sample: restricted to NHW, US-based respondents with complete and adequate test data and county data. Sensitivity sample: restricted to NHW, US-based respondents with complete and adequate test data, complete county data, and taking the IAT for the first time (excludes those with num_tests > 0 OR num_tests = NA).

### Appendix E.3. Construction of Non-Hispanic White

Our analysis is focused on the racial bias of non-Hispanic White Project Implicit respondents. Therefore, we included all White respondents identifying as “not Hispanic or Latino” and excluded those identifying as White and “Hispanic or Latino.” However, a small proportion of White respondents indicated “unknown” ethnicity or did not report an ethnic identity. These respondents were retained in the final sample under the assumption that non-Hispanic/Latino individuals would be more likely to report ethnicity = ”unknown” or leave the question blank than those identifying as Hispanic/Latino. Thus, the sample was restricted to respondents who identified as White and who did not explicitly indicate ethnicity is “Hispanic/Latino.” Those identifying as White for whom ethnicity was missing or unknown (n = 65,533) were assigned non-Hispanic White and retained in the county-averages (n = 723,271). As a sensitivity analysis, we excluded White respondents with unknown or missing ethnicity, retaining only those who explicitly reported ethnicity = “Not Hispanic or Latino” (n = 657,738). Basic demographics and racial bias scores between non-Hispanic White respondents under the two coding approaches were nearly identical.

**Table A8:** Characteristics of IAT test-takers by construction of non-Hispanic White, 2017–2019.

Characteristic	Primary Coding (n = 723,256)	Sensitivity Coding (n = 657,727)
Gender identity	n (%)	n (%)
Woman	451,908 (62.5)	414,745 (63.1)
Man	262,992 (36.4)	235,520 (35.8)
Nonbinary	7939 (1.1)	7106 (1.1)
NA	417 (0.1)	367 (0.1)
Age (mean (SD))	29.7 (13.9)	29.9 (13.8)
Educational attainment		
<high school	97,418 (13.5)	80,968 (12.3)
high school graduate	283,493 (39.2)	255,932 (38.9)
bachelor’s degree	340,940 (47.1)	319,718 (48.6)
Missing	1405 (0.2)	1120 (0.2)
Number previous tests		
0	477,404 (66.0)	429,692 (65.3)
1	143,625 (19.9)	132,160 (20.1)
2	52,608 (7.3)	49,065 (7.5)
3–5	39,000 (5.4)	36,838 (5.6)
6 +	9861 (1.4)	9397 (1.4)
Missing	758 (0.1)	586 (0.1)
Implicit racial bias (mean (SD))	0.36 (0.41)	0.36 (0.42)
Explicit racial bias (mean (SD))	0.24 (1.54)	0.25 (1.53)

Project Implicit sample restricted to NHW, US-based respondents with complete and adequate test data and county data. Primary coding: Restricted to respondents who identified as White and who did not indicate ethnicity is “Hispanic/Latino.” Includes those identifying as White for whom ethnicity was missing or unknown (n = 65,533), Sensitivity coding: Restricted to respondents who identified as White and explicitly stated ethnicity = not Hispanic. Excludes those identifying as White for whom ethnicity was missing or unknown (n = 65,533).
